# Osteoarthritis: quality of life, comorbidities, medication and health service utilization assessed in a large sample of primary care patients

**DOI:** 10.1186/1749-799X-2-12

**Published:** 2007-06-30

**Authors:** Thomas Rosemann, Gunter Laux, Joachim Szecsenyi

**Affiliations:** 1 Department of General Practice and Health Services Research, University of Heidelberg, Voßstrasse 2, 69115 Heidelberg, Germany

## Abstract

**Objective:**

To assess the gender related impact of osteoarthritis (OA) on quality of life (QoL) and health service utilization (HSU) of primary care patients in Germany.

**Methods:**

Cross sectional study with 1250 OA patients attending 75 primary care practices from March to May 2005. QoL was assessed using the GERMAN-AIMS2-SF. Data about comorbidities, prescriptions, health service utilization, and physical activity were obtained by questioning patients or from the patients' medical files. Depression was assessed by means of the Patient Health Questionnaire (PHQ-9).

**Results:**

1021 (81.7%) questionnaires were returned. 347 (34%) patients were male. Impact of OA on QoL was different between gender: women achieved significantly higher scores in the AIMS 2-SF dimensions lower body (p < 0.01), symptom (p < 0.01), affect (p < 0.01) and work (p < 0.05). Main predictors of pain and disability were a high score in the "upper body "scale of the AIMS2-SF (beta = 0.280; p < 0.001), a high score in the PHQ-9 (beta = 0.214; p < 0.001), duration of OA (beta = 0.097; p = 0.004), age (beta = 0.090; p = 0.023) and the BMI (beta = 0.069; p = 0.034). Predictors of pain and disability did not differ between gender. 18.8 % of men and 19.7% of women had a concomitant depression. However, no gender differences occurred. Women visited their GP (mean 5.61 contacts in 6 months) more often than men (mean 4.08; p < 0.01); visits to orthopedics did not differ between gender.

**Conclusion:**

The extent to which OA impacts men and women differs in primary care patients. This might have resulted in the revealed differences in the pharmacological treatment and the HSU. Further research is needed to confirm our findings and to assess causality.

## Background

Osteoarthritis is one of the most prevalent chronic diseases worldwide and is associated with substantial impact on patients' individual quality of life as well as on healthcare costs. Its prevalence is expected to rise significantly in the upcoming decades. Increasing life expectancy and decreasing physical activity, leading to a constant increase in body weight, are regarded as underlying determinants of this development. Facing this situation, the WHO and the United Nations have declared the years 2000 to 2010 to be the "Bone and Joint Decade" [1]. Since in the year 2050 more than 50% of the population will be over 50 years of age, the German health care system will be hit tremendously by chronic illnesses like osteoarthritis [2]. Most of these individuals will receive medical treatment in primary care settings, accounting for the growing number of studies dealing with OA in primary care [3-5]. However, to date, relatively little is known about osteoarthritis symptoms and their medical treatment in various subgroups of patients in primary care.

Previous studies have focused on the prevalence and prognosis of OA [4,6]. Regarding prevalence, it is a frequently replicated result that women have a higher probability for developing OA, especially OA of the knee [7,8]. Several studies have suggested that not only prevalence but also the disease process is related to gender: women were found to have more severe structural progression and a higher need to undergo surgical interventions than men [6]. Other studies suggested that women with OA suffer from pain and disability to a greater extent compared to men and also that these dimensions of QoL are strongly associated with the social situation [9-11]. However, it remains unclear how these findings can be explained.

The present study was performed to get a comprehensive overview of the health status and the healthcare received by primary care patients with OA in Germany. We particularly focused on differences related to gender because we hypothesized that men and women differ regarding health status and health service utilization (HSU). Furthermore, since it is known that quality of life (QoL) of OA patients is mainly determined by pain and disability our aim was to assess factors that are associated with these two dimensions of QoL [8,12].

## Materials and methods

The data used for this study are retrieved from the baseline assessment of the PraxArt project, which is financed by the German Ministry for Education and Research over a period of 6 years, starting in 2003. The aim is to assess the status of OA care and to search for possibilities to improve care as well as patients' quality of life by tailored interventions. A randomly created sample of 75 general practitioners in the area of Baden-Wuerttemberg and Bavaria has been enrolled and recruited the patients for this survey.

### Participants

To be eligible for inclusion, patients had to be adult and diagnosed with osteoarthritis of the hip or knee according to the Committee of the American Rheumatism Association[13,14]. In each of the participating 75 practices, 15 patients fulfilling these criteria were addressed consecutively. In total, 1250 questionnaires were administered to patients after they had given their written informed consent. They were asked to return the questionnaires to the university by sending a stamped envelope and were informed that neither the GP nor the practice team had any possibility to get knowledge of their answers. GPs created a list of all addressed patients. Since the patients were addressed by their GP, detailed information about sociodemographic data, comorbidities, and medication were also available for the non-respondents.

### Data collection

Sociodemographic data included gender, age, educational level (1 = no school degree to 5 = university degree), working situation (1 = unemployed or retired, 2 = half time, 3 = fulltime), and partnership (1 = living alone, 2 = married/living with partner). As in the long version of the AIMS2, some important comorbidities were assessed in the questionnaire: high blood pressure (HBP), diabetes, heart insufficiency (HI), coronary vessel disease (CVD), elevated cholesterol level (low density lipoprotein (LDL) > 200 mg/dl), ulcer or stomach disease, asthma/chronic obstructive pulmonary disease (COPD), kidney disease, cancer and stroke. Patient's answers were compared with comorbidities mentioned in the medical file via the list the GPs had created when addressing patients. This was done to increase validity of data and also to assess accuracy of self reported diagnosis later on in the project. The same procedure was performed for all other answers, including disease duration. For the analysis in this study the date form the medical files were used. Disease duration was defined as the period form mentioning OA for the first time in the medical file till now. Depressive disorder was diagnosed using the depression module of the German form of the Patient Health Questionnaire (PHQ-9) [15]. The PHQ-9 is a self-administered questionnaire that enables to diagnose a Major or Minor Depression Episode according to DSM-IV [16,17]. Moreover, the summarized scale score allows assessing the severity of depression. The PHQ-9 has proven to be a valid instrument for these assessments [18,19].

The impact of OA on patients' health was assessed by the GERMAN-AIMS2-SF, which provides a comprehensive assessment of patients' health status comprising the dimensions physical limitation, symptom (reflecting perceived pain), social (reflecting social contacts), affect (reflecting mood), and work (reflecting the ability to work). It has recently been validated in German language in a sample of OA patients [20]. As suggested in this study, we divided the physical limitation scale of the AIMS2-SF into upper body limitation and lower body limitation. To get a comprehensive view of the present situation of OA patients, we collected all information about medication and HSU from the patients' files. Since not all information on medication (e.g. OTC medication), HSU (e.g. visits to healers) and treatments (e.g. acupuncture) were available in the files, we assessed data about these parameters by straightforward questions. As described above, to complete data, each questionnaire was compared with the medical file to which it was linked by an identification number on the participants list. So, data given by patients could be checked by comparing them with the medical file. Non-respondents were identified by comparing GPs lists of addressed patients with received questionnaires.

Patients were asked to mention all disease specific medications they take additionally to prescriptions, including OTC medication, homeopathic medication and symptomatic slow-acting drugs in osteoarthritis (SYSADOA). SYSADOA is a generic term and covers a wide range of substances. In Germany some of them have to be prescribed, others are regarded as health food supplement, as it is the case for instance in the United Kingdom. We assessed separately whether SYSADOAs were prescribed or whether patients bought them without prescription. Regarding Health Service Utilization (HSU), patients were asked about all contacts to orthopedic surgeons, healers, received x-rays, physiotherapy, acupuncture and intraarticular injections. The International Physical Activity Questionnaire (IPAQ), a widespread assessment instrument was used to assess physical activity [21]. Inclusion of patients did not start unless there was a written and unrestricted positive vote of the ethics committee of the University of Heidelberg which was received in March 2005 (approval number 021/2005).

### Statistical analysis

The data were analyzed with SPSS (version 12.0). Descriptive analyses were performed for all variables. Continuous variables are reported using means, standard deviations (SD), ranges and percentages. Unadjusted group comparisons were performed by means of Student's t-test. Normality was tested by means of Kolmogorov-Smirnov-test to allow parametric test were applicable. For the comparison of medication, comorbidities and depression categories, which represented binary variables, the Chi-square-test was used. Since the prevalence of depression differs between men and women, the analysis was performed separately for gender[22]. Comparisons of depression prevalence (PHQ-scores), and HSU were made by ANCOVAs adjusted for covariates that may have substantial influence such as age, disease duration, comorbidities and QoL (AIMS2-SF scales assessing pain, physical limitation and social). Pain and disability are known to be the most important factors determining QoL in OA patients. To assess predictors of these two factors, we calculated a sum score of the AIMS "symptom" and "lower body" dimension and calculated univariate correlations to sociodemographics and disease characteristics (by means of Spearman's rho). Factors with significant correlations were included in a stepwise regression analysis (method: enter) to reveal significant predictors.

## Results

In total, 1311 patients were addressed by the GPs. 1250 of them agreed to complete the questionnaire. 1021 of the 1250 (81.7%) patients returned the questionnaires, corresponding to at least 11 questionnaires in each practice. Regarding available data, including sociodemographic variables, comorbidities and medication, no statistically significant differences could be revealed between the non-respondents and the respondents. The main reason given for not participating was time effort. Among the enrolled patients, 347 (34.0%) were male and 674 (66.0%) were female. If missing data occurred, they mainly occurred within the same questionnaire, in total in 271 of the 1021 questionnaires. In 123 cases the data could be completed from the patient file.

278 (80.1%) men were married or lived with a partner. 376 (55.8%) women were engaged. This difference was significant (p < 0.01). Completely retired from work were 233 (67.1%) men and 482 (71.5%) women. T-test for group comparison revealed a significant difference in the (formal) educational level between men (mean 2.61, SD 1.1) and women (mean 2.38, SD 0.83). BMI, age, number of comorbidities or disease duration did not differ significantly. Table 1 displays the characteristics of the study sample separated by localization of OA.

**Table 1:** Characteristics of enrolled patients (n = 1021) separated by localization of OA

	total	Hip		Knee	
		Men	Women	Men	Women

Number of participants	1021	191	236	156	438
Mean (SD) age (y)	66.1 (15.1)	64.3 (14.8)	65.3 (14.5)	66.5 (15.4)	67.1 (15.4)
Disease duration (years)	13.7 (13.0)	11.7 (10.8)	12.3 (12.1)	14.1 (12.9)	15.2 (14.2)
Body mass index (kg/m^2^)	28.3 (4.7)	27.4 (3.7)	27.1 (4.5)	28.6 (3.4)	29.2 (4.8)

AIMS2-SF dimensions:					

Lower body **	2.64 (2.04)	2.19 (1.97)	2.22 (2.25)	2.81 (2.71)	3.01 (2.95)
Upper body	1.21 (2.02)	1.02 (1.86)	1.19 (1.17)	1.09 (1.77)	1.34 (2.32)
Symptom **	4.71 (2.42)	4.21 (2.13)	4.37 (2.19)	4.68 (2.83)	5.12 (2.91)
Affect **	2.77 (1.51)	2.39 (1.42)	2.81 (1.48)	2.52 (1.50)	3.01 (1.59)
Social	4.57 (1.95)	4.26 (1.88)	4.44 (1.72)	4.45 (1.90)	4.82 (2.03)
PHQ-9 sum score	15.7 (4.69)	14.9 (4.33)	15.9 (4.86)	15.4 (5.02)	16.1 (5.32)
Unilateral OA (%)	150 (13.8)	32 (16.7)	41 (17.3)	32 (20.5)	45 (10.2)
Number (%) with bilateral OA	871 (84.5)	159 (83.2)	195 (82.6)	124 (79.5)	393 (89.7)
Number (%) with generalized OA^¶^	282 (25.4)	26 (13.6)	57 (24.1)	33 (21.1)	166 (37.8)

### Quality of Life

Regarding the impact of the disease on QoL, women achieved significantly higher scores in the lower body scale (2.98 vs. 2.39; p < 0.01), indicating more physical disability. Also the scores in the affect scale (3.10 vs. 2.60; p < 0.01) and the symptom scale (5.12 vs. 4.49; p < 0.01) indicated that women had significantly lower mood and significantly more perceived pain than men (Table 1). This result remained significant even if the ANCOVAs were adjusted for age, disease duration and comorbidities. Due to the inclusion criteria, focusing on patients with OA to the lower limb, the scores for the upper body limitation were low, indicating no functional disability and did not differ by gender. Differences in the work scale were notable (p < 0.05), but because of the large number of retired patients the absolute numbers were small.

### Comorbidities

Table 2 displays the distribution of comorbidities separated by gender. As can be seen, high blood pressure and elevated cholesterol were the most common comorbid conditions. Significant gender differences occurred only regarding HBP (p < 0.01). Asked about side effects related to their osteoarthritis medication, 282 (81.27%) men and 563 (83.53) women agreed to have had side effects during the last 6 months. Interestingly, 77 men (22.19 %) and 146 women (21.66%) reported having ulcer or stomach pain in their history.

**Table 2:** Comorbidities of the study sample (n = 1021) separated by gender

gender	High blood pressure**	Elevated cholesterol	Diabetes	Heart Insufficiency	Coronary vessel disease	Ulcer/Gastritis	Asthma/COPD	Renal Insufficiency	Cancer	Stroke
male	181	124	57	63	62	77	34	23	21	16
%	52.1	35.7	16.4	18.1	17.8	22.1	9.8	6.6	6.1	4.6
female	384	245	120	131	70	146	64	33	16	30
%	56.9	36.3	17.8	19.4	10.3	21.6	9.5	4.9	2.4	4.4
Total %	55.2	36.1	17.3	19.0	12.9	21.8	9.6	5.5	3.6	4.5

* p < 0.05; ** p < 0.01 in Chi-square test

### Depression as comorbidity

In summary, 344 (99.1%) men and 668 (99.1%) women completely answered all 9 items of the PHQ-9 (table 3). Among these, 38 (11.0%) men fulfilled criteria for a major depressive episode and 27 (7.8 %) fulfilled criteria for a minor depressive episode. In women, 84 (12.6 %) had a Major Depression episode and 47 (7.1 %) a Minor Depression episode. The overall prevalence of depressive disorders was 19.4%. ANCOVA adjusted for age, disease duration and comorbidities revealed no significant differences in PHQ-9 scores as well as in the occurrence of minor and major depression between men and women. Additionally, a Chi-square test was performed to compare the severity categories as binary data (no depression, minor, major). This test also revealed no gender difference.

**Table 3:** Scores of the severity index of depression (PHQ-9 questionnaire)

		PHQ-9 scores		Fulfilling criteria for		Overall
		
Gender	N	Mean	SD	Major Depression	Minor Depression	Depressive Disorder
male	344	15.33	4.76	38 (11.0%)	27 (7.8 %)	65 (18.9%)
female	668	15.95	4.63	84 (12.6 %)	47 (7.1 %)	131 (19.6%)
∑				122 (12.0%)	74 (7.3%)	196 (19.4%)

* p < 0.05; **p < 0.01; PHQ-9 scores compared by ANCOVA (adjusted for age, disease duration and comorbidities); severity categories by means of Chi-square-test

### Health Service Utilization

Table 4 displays the health service utilization of the study sample within the last 6 months before the assessment. 86.4% of women and 76.7% of men visited their GP at least once during the last half year. The amount of visits to the GP varied widely from 0 to 12 during half a year with a mean of 5.61 (SD 8.26) in women and 4.08 (SD 6.29) in men, representing a significant difference (p = 0.001) in ANCOVAS adjusted for age, disease duration and comorbidities. Regarding visits to orthopedic surgeons, with a mean of 1.88, women had slightly more contacts than men (mean 1.68), but the difference remained not statistically significant after adjusting for covariates as mentioned above. More than a quarter of the patients received some acupuncture during the last half year and nearly a quarter visited a traditional healer at least once. ANCOVAs revealed that men received significantly more often injections in the joint (p = 0.026), but less acupuncture (p = 0.042). Regarding physiotherapy, performed x-rays, and visits to healers, no significant differences between gender could be revealed by means of ANCOVAs.

**Table 4:** Health service utilization of the study sample within 6 months

During the last 6 months	Gender	At least one (%)	Mean	SD
GP visits **	Male	76.7	4.08	6.29
	Female	86.4	5.61	8.26
Visits to Orthopedic surgeon	Male	56.8	1.68	3.17
	Female	58.8	1.88	3.77
Physiotherapy*	Male	51.3	5.66	11.52
	Female	60.5	7.26	12.08
X-rays of joint	Male	49.1	0.78	3.82
	Female	52.5	0.98	4.15
Intraarticular injections**	Male	34.3	1.20	4.38
	Female	30.6	0.89	4.08
Acupuncture*	Male	26.8	0.68	2.69
	Female	27.9	1.22	4.53
Visits to Healers	Male	22.5	0.21	1.31
	Female	23.5	0.33	3.21

* p < 0.05, ** p < 0.01 in adjusted ANCOVA (age, disease duration, comorbidities)

### Pharmacological treatment

NSAIDs represented the most frequently prescribed medication in our study sample. Women received NSAIDs significantly more often (p = 0.043) than men, while the gender differences in the less often prescribed COX-2-inhibitors were not significant (Table 5). Paracetamol was assessed because it is recommended as treatment of first choice in most guidelines. Interestingly, Paracetamol was used only marginally. About 5% of patients of each gender were treated with opiates, women received significantly more opiates that belonged to step III according to the WHO step scheme of pain treatment. Overall, SYSADOAs were only marginally prescribed, they were mostly taken as OTC medication. Regarding homeopathic medication, no significant difference could be observed between gender.

**Table 5:** Medication of the study sample (n = 1021) separated by gender

	Pain relievers						Homeopathics	SYSADOA (OTC)**	SYSADOA (prescription)	Muscle relaxant
					
	NSAID		Opiats		others	Paracetamol				
							
	Unselective COX-inhibitors*	COX-2	WHO II	WHO III						
Male (347)	120 (34.6%)	8 (2.3%)	18 (5.2%)	4 (%1.15)*	7 (2.0%)	2 (0.6%)	20 (2.0%)	34 (9.8%)	9 (2.6%)	8 (2.3%)
Female (674)	276 (40.1%)	18 (2.7%)	32 (4.8%)	14 (2.0%)*	14 (2.0%)*	8 (1.2%)*	49 (2.5%)	78 (11.6%)	18 (2.7%)	14 (2.1%)
∑	38.7%	2.6%	4.9%	1.8%	2.1%	1.0%	6.8%	10.7%	2.64	2.2

* p < 0.05; **p < 0.01 in Chi-square-test

Table 6 displays univariate correlations between the sum score of the "symptom" and the "lower body" scale of the AIMS2-SF reflecting the main impact of arthritis on QoL, pain and disability. Factors which achieved significance were entered into the regression model. As can be seen in table 7, the main predictors of "pain and disability" are a high score in the "upper body "scale of the AIMS2-SF (beta = 0.280; p < 0.001), a high score in the PHQ-9 (beta = 0.214; p < 0.001), duration of OA, age and the BMI. The whole model explained over 40% of variation in the dependent variable. In a first approach the analyses was made separately for both gender. Interestingly, predictors were the same for both gender, differences occurred only with respect to the amount of the regression coefficient beta. Consequently, the results were displayed for both gender together.

**Table 6:** Correlations between sociodemographic and disease characteristics with "pain and disability"

	Spearman's' rho	p
Sociodemographics		
Age	0.052	0.129
Marital status	-0.069	0.028
Gender	0.096	0.002
Education	-0.076	0.016
IPAQ sum score	-0.033	0.346
BMI	0.157	<0.001
Health service utilization		
GP contacts	0.251	<0.001
Visits to Orthopedics	0.238	<0.001
Visits to healers	0.007	0.831
Amount of performed X-rays	0.254	<0.001
Physiotherapy	0.207	<0.001
Amount of prescriptions	0.178	<0.001
Disease characteristics		
Duration of OA	0.269	<0.001
Amount of comorbidities	0.221	<0.001
PHQ-9 sum score	0.475	<0.001
Quality of life/AIMS2-Sf scales		
Upper body	0.398	0.001
Affect	0.472	0.001
Social	0.212	<0.001
Work	-0.018	0.569

**Table 7:** Linear Regression analysis, dependent variable: "pain and disability"

R^2^= 0.425; adjusted R^2 ^= 0.402 F = 18,12; p < 0.001	Regression coeffizient beta	T	p
Upper body*	0.280	7.978	<0.001
PHQ-9 sum score	0.214	4.817	<0.001
Duration of OA	0.097	2.923	0.004
Age	0.090	2.280	0.023
BMI	0.069	1.928	0.034

* AIMS2-SF scale

## Discussion

Based on previous findings indicating biological differences (e.g. regarding the destruction of the cartilage) and psychological differences (e.g. perception of pain) we hypothesized that men and women differ regarding many aspects of QoL and received care. This hypothesis could be confirmed: OA has higher impact on women in important aspects of QoL such as pain, disability and mood. Similar gender differences have been found e.g. by Woo et al. among Chinese people [23]. They received more NSAIDs and visited their GP but not their specialist more frequently than men and tended to have less intraarticular injections. Interestingly, minor or major depressive episodes were not more frequent among women, even though the affect scale of the AIMS2-SF indicated lower mood among women.

Regarding QoL, we found lower scores than Sany et al. did in a sample of rheumatoid patients regarding physical limitation. However, we observed nearly the same mean scores regarding the symptom scale. This finding may indicate that patients suffering from OA are less limited in their mobility but appear to suffer from equivalent pain intensity than patients with rheumatoid arthritis (RA). With regard to comorbidities which have an important impact on the QoL of patients suffering from osteoarthritis as well as on the outcome of surgical interventions, gender differences occurred only regarding high blood pressure [24-27]. Unfortunately, reliable data regarding comorbidities in OA patients are difficult to compare since different comorbid conditions have been assessed with different methods (e.g. self reports) in previous studies. Groessl et al. who enrolled 363 OA patients in a primary care setting in a health management organization (HMO) in the United States reported on somewhat lower rates of HBP (28.8 %), which was the commonest comorbidity in their sample. Similar numbers were found by Nilsdotter et al. [26]. Compared to national data, the prevalence of HBP in Germany in this age group is expected to be over 55%, as was found in a large international comparison [28]. However, a limitation of our findings is that no control group was available.

Regarding pain medication, Paracetamol, which is the first choice treatment according to most guidelines, was only marginally prescribed. The main pillar in pharmacological treatment are NSAIDs such as Diclofenac [29-31]. This is in accordance with the fact that NSAIDs are known to be increasingly used worldwide [32]. Interestingly, COX-2-inhibitors played no important role in prescriptions. Our data also confirmed previous findings showing that the use of NSAIDs is more frequent among women than men [33]. In the study of Linsell et al. 45.9% of OA patients stated to take pain killers frequently, which is comparable to our results[3].

Regarding HSU, our data indicated a high HSU by OA patients. However, it has to be noted that the German health care system is characterized by a high physician contact-rate. The number of mean contacts per year and person in Germany, including all contacts to GPs and specialists, is 6.6 [34]. In Germany patients have free access to secondary care, a referral is not required [35]. Thus, the revealed high amount of x-rays for example may also be due to the unlimited accessibility of health care in Germany [36]. The reason why women visited their GP more often than men could be related to the higher pain scores of women, since it is known that pain is a strong predictor for HSU among OA patients [27]. Interestingly, gender differences could only be revealed regarding contacts to GPs but not to specialists. An important weakness of the presented data is that, even though the analyses were adjusted for important covariates, HSU may have been related to other reasons except arthritis, even though we asked patients to mention only contacts which were related to OA. It should also be mentioned that we did not control our data for patients' insurance. About 10% of patients are "privately" insured, resulting in higher reimbursement for physicians. This may have influence on treatments, prescriptions as well as on referral rates. Our data regarding HSU reflect a finding that may be ignored by many physicians: the important role of complementary alternative medicine (CAM) for patients with OA. As Rao et al. could show the use of CAM is very common among patients with RA. Our data regarding visits to healers and received acupuncture are lower in comparison to the findings of Rao, who reported a frequent use of up to 90% among the RA patients, but at least more than a quarter of our patients reported on current use of CAM [37]. According to Rao, only half of the patients discuss the use of CAM with their physician, so they should be aware of this issue and address it in order to avoid treatment conflicts or side effects. Interestingly, comparable findings regarding CAM have been reported by Linsell et al. in a sample of OA patients in the UK [3]. Many studies have assessed depression in patients with rheumatoid arthritis, some of which indicate a higher risk among patients with RA than OA patients [38,39]. None of them enrolled as much OA patients as we did. The importance of depression for OA patients is related to the fact that it is an important predictor for functional disability and an independent risk factor for mortality in RA [40]. Previous findings regarding the prevalence among OA patients indicated no increased prevalence [41,42]. Our data showed that 19.7% of women and 18.9% of men fulfilled the criteria for a major or minor depressive episode. Data regarding the point prevalence among the German population vary between 5–10 % in the general population [19]. In contrast to the general population, no gender differences could be revealed in our study sample. Our findings indicate a significant increase in the point prevalence but the numbers of about 30% reported for RA patients were not met [43].

Pain and disability have often been shown as the major burden of OA. Similar as in various other studies, women achieved higher scores regarding both symptoms of OA [23]. But interestingly, no gender differences could be revealed regarding their predictors.

Despite the fact that our study has certain limitations and acknowledging the characteristics of the German health care system with e.g. a large number of non-surgical orthopedics, the study gives a comprehensive overview. However, because of the wide range of aspects addressed in this paper, it is not possible to describe the findings in detail e.g. in the sense of revealing predictors for each variable. The study represents the largest assessment of OA patients in a primary care setting in Germany.

Our findings regarding QoL and the burden of the disease suggest that OA patients differ from patients suffering from other forms of arthritis, especially RA. Our findings suggest that the impact of OA on men and women differs. Even we could not prove causality we assume that this may be have lead to the revealed differences in the pharmacological treatment and the use of the health care system. Further research is needed to confirm our results and assess causality.

## Competing interests

The author(s) declare that they have no competing interests.

## Authors' contributions

TR conceived and performed the study and drafted the manuscript. GL performed the data management and statistical calculations. JS participated in the study design. All authors read and approved the final manuscript.
